# Tailoring of Self-Management Interventions in Patients With Heart Failure

**DOI:** 10.1007/s11897-015-0259-3

**Published:** 2015-05-02

**Authors:** Irene Bos-Touwen, Nini Jonkman, Heleen Westland, Marieke Schuurmans, Frans Rutten, Niek de Wit, Jaap Trappenburg

**Affiliations:** Department Rehabilitation, Nursing Science & Sports, University Medical Center Utrecht, PO Box 85500, 3508 GA Utrecht, The Netherlands; Julius Center, Department of General Practice, University Medical Center Utrecht, PO Box 85500, 3508 GA Utrecht, The Netherlands

**Keywords:** Heart failure, Self-management interventions, Tailoring, Patient characteristics

## Abstract

The effectiveness of heart failure (HF) self-management interventions varies within patients suggesting that one size does not fit all. It is expected that effectiveness can be optimized when interventions are tailored to individual patients. The aim of this review was to synthesize the literature on current use of tailoring in self-management interventions and patient characteristics associated with self-management capacity and success of interventions, as building blocks for tailoring. Within available trials, the degree to which interventions are explicitly tailored is marginal and often limited to content. We found that certain patient characteristics that are associated with poor self-management capacity do not influence effectiveness of a given intervention (i.e., age, gender, ethnicity, disease severity, number of comorbidities) and that other characteristics (low: income, literacy, education, baseline self-management capacity) in fact are indicators of patients with a high likelihood for success. Increased scientific efforts are needed to continue unraveling success of self-management interventions and to validate the modifying impact of currently known patient characteristics.

## Introduction

Heart failure (HF) is a major health problem [1, 2], characterized by a wide spectrum of debilitating symptoms, poor quality of life, frequent hospitalizations, and high mortality [3, 4]. Outcomes have improved over the last two decades for those with HF and a reduced ejection fraction with pharmacological therapy, devices, and multidisciplinary management programs [3, 5].

A part of the progression of HF and its burden on patients and society is thought to be preventable if patients are engaged in the (proactive) management of their disease [6, 7]. Patients who are actively involved in their care and who have the competences to adhere to treatment regimens and take appropriate actions are expected to have better survival and reduced hospital (re-)admissions [8–10]. In the literature, the terms self-management, self-maintenance, and self-care sometimes are used as separate terms [11] and sometimes used interchangeably to cover the complex challenges patients face in coping with their disease [12]. To enhance the readability and to remain consistent with international chronic care models [13], we will use the term self-management throughout this review. The key targeted HF self-management competences are (i) adherence to complex medication regimens, (ii) monitoring symptoms and taking appropriate action when needed, and (iii) compliance with exercise, dietary, and other lifestyle recommendations [14].

Over the past decades, many interventions have been developed and evaluated that may help to equip patients with these complex self-management competences. Most interventions provided (nurse-led) patient education and training skills to support self-management [15]. Several meta-analyses have highlighted the heterogeneity in delivery, methods, and duration of these interventions. Reviews including studies that evaluated isolated educational interventions showed improvements in knowledge [16, 17]. When self-management was studied as part of more comprehensive disease-management or telehealth program, more robust results were reported, such as reduction of disease-related hospital admissions [18–23], mortality [18, 19, 22], and improved health-related quality of life (HRQoL) [24]. One meta-analysis calculated the pooled results of isolated self-management interventions and found a reduction in HF and all-cause hospitalizations and improved medication adherence but not in mortality, HRQoL, functional capacity, or symptoms [25]. The results from the aforementioned reviews indicate that the evidence for effectiveness of self-management interventions is ambiguous. This may be partly due to mixed quality of the reviews [26], heterogeneity in program and patient characteristics [27], and low treatment fidelity of the provider [28].

There is a need to better understand the key elements of successful self-management interventions. An important starting point is recognizing that managing a complex multi-impact disease is difficult. This is supported by several studies showing that self-management capacity in patients with HF is generally poor [29–32]. Pooled results from 15 countries indicated that poor uptake of self-management is a worldwide phenomenon [33••]. Besides variations in self-management capacity, longitudinal studies have shown that patient-specific characteristics also seem to modify the effectiveness of self-management interventions. Meta-analytical findings have indicated that a substantial number of patients do not or insufficiently respond to self-management interventions [25, 34]. The current premise that some interventions work better in specific patient populations and not in others suggest that one size does not fit all [27, 35•]: in other words, more tailored approaches will be needed.

In a tailored approach, the treatment exposure is dynamic instead of the more fixed exposure in one-size-fits-all interventions [36]. In a tailored intervention, the individual is assessed and the intervention is customized based on the unique characteristics of that person, in order to increase the relevance of treatment and to produce greater desired changes [37]. Customization refers to the personalized treatment consequence, such as variations in content (topics), behavior change techniques, mode of delivery, and dose [38]. A specific type of tailoring is targeting in which subgroup characteristics are used to develop a single intervention for a defined population subgroup [36].

To optimize tailoring of interventions in HF, a thorough understanding of effective intervention ingredients is required, as well as knowledge of tailoring strategies and subgroups of patients in which a given self-management intervention is most effective. The aims of this review are (1) to describe the current use of tailoring in self-management interventions in patients with HF and to synthesize the essential literature on patient characteristics associated with (2) self-management capacity and (3) intervention effects.

## Current Use of Tailored Self-Management Approaches in Clinical Trials

Recently, we established a large international collaboration between principal investigators of HF self-management trials [35•]. The aim of this consortium is to unravel success of self-management interventions through pooling and modeling individual patient data (IPD) in a meta-analysis of previously conducted randomized clinical trials. The search and selection strategy applied in the IPD meta-analysis has been described in detail elsewhere [35•]. Self-management interventions were operationalized as patient-focused interventions with at least two of the following components: (1) active stimulation of symptom monitoring, (2) education in problem solving skills (i.e., self-treatment such as managing acute exacerbations, resource utilization, and stress/symptom management), and enhancement of either (3) medication adherence, (4) physical activity, (5) dietary intake, or (6) smoking cessation. For this review, we used a similar search strategy of the ongoing IPD meta-analysis and updated the study selection (until January 2015) [35•].

Data on presence and type of tailoring were extracted for 52 self-management interventions. Tailoring was considered present if the intervention was personalized, and based on an assessment of individual characteristics. Table 1 provides an overview of the tailoring strategies in HF self-management interventions and the theory underlying the tailored intervention. Of the 52 selected studies, 28 explicitly described their tailoring strategy. The sample size of the studies ranged from 40 to 715, and the majority were nurse-led interventions. Studies not included in Table 1 were either not tailored or the investigators did not explicitly report their tailoring strategy [39–45, 46••, 47–62]. This includes studies in which the content might have been individualized to some extent but no clear description was provided on how this was applied. For example, in interventions with face-to-face consultations, interpersonal communication, goal setting, and problem solving could have resulted in individualization of the intervention, but this was not clearly reported in the publication.

**Table 1:** Characteristics of tailored interventions in heart failure (HF) self-management trials

Author, year	Number	Intervention	Tailoring variables^a^	Customization variables			Theory underlying intervention
				Content/ themes	Mode of delivery	Dose/frequency	
Angermann et al. [72] 2012	715	Nurse-led telephone contact intervention during 6 months	Knowledge, needs, NYHA class	X		X	NR
Atienza et al. [106] 2004	338	Nurse-led intervention with 1 session prior to discharge, tele-monitoring, a visit with PCP and 3 monthly follow-up visits to cardiologist (during 1 year)	Knowledge of disease, ability to identify signs and symptoms of heart failure worsening, response to deterioration, patients’ performance	X			NR
Barnason et al. [67] 2010	40	Nurse-led intervention with 1 face-to-face and 2 telephone contacts	Health literacy, medication adherence, medication perceived needs	X	X		Social cognitive and learning theory and the medication adherence conceptual framework
Clark et al. [68] 2014	50	Nurse-led intervention with 3 months of biweekly individual sessions followed by 3 months of TCs/e-mails	Interest of the participant		X	X	Based on a conceptual model by Stuifbergen et al. (combination of health promotion theory and self-efficacy theory)
Cockayne et al. [107] 2014	260	Nurse-led intervention with 6 individual sessions	Cardiac misconceptions, discussion of patient’s medication and of the patient’s risk factors	X			Not described, but use of cognitive behavioral strategies
Davis et al.[74] 2012	125	Case manager-led intervention during hospitalization and 1 TC 1–3 days after discharge	Patient’s personal routine and environment	X			NR
DeWalt et al.[75] 2006	605	Health educator-led intervention with individual session and TC FU for 12 months	Patient’s knowledge and interests	X			NR
DeWalt et al.[63••] 2012	605	Health educator-led intervention with individual session and TC FU for 12 months	Patient’s knowledge, behaviors and language preference	X	X	X	The social cognitive theory and learning theory
Dickson et al.[64] 2014	75	Lay health educator led intervention of twice-weekly, 1 h group session for 4 weeks	Knowledge, skills, circumstances and needs	X	X		The situation-specific theory
Dracup et al.[108] 2014	602	Nurse-led intervention with 2 intervention arms of 1 individual session and per arm different frequencies of TC FU	Medication list, barriers, content competency	X			NR
Dunagan et al.[69] 2005	151	Nurse-led telephone intervention with telephone contact and/or 1 home visit	Patients’ clinical status and self-management abilities			X	NR
Dunbar et al.[109] 2013	170	Nurse-led intervention of 2 (group) sessions in 2 months and the second arm additional 2 sessions (+2 months).	Perceptions of living with HF; sodium intake and medication adherence; dyads autonomy support and family criticism scores	X			Self-determination theory
Ekman et al.[70] 1998	158	Nurse-led intervention with telephone and/or individual contacts up to 6 months	Understanding of disease and treatment and relevant psychosocial and lifestyle factors	X		X	NR
Jurgens et al.[110] 2013	99	Primary investigator (nurse)-led intervention of 2 individual sessions within 10 days	Sensations of dyspnea and fatigue	X			Theory of heart failure self-care
Leventhal et al.[111] 2011	42	Nurse-led intervention with one individual session and FU with 17 TCs	Physical, psychosocial and environmental assessment	X			Not clearly described but a component of the self-efficacy theory is included
Jaarsma et al.[112] 1999	179	Nurse-led intervention with a TC of 1 week and a visit 10 days after discharge	Patients’ needs and potential problems	X			NR
Otsu et al.[113] 2011	96	Nurse-led intervention of 6 individual sessions during 6 months	Sodium use, level of compliance and performance at home	X			Theory of cognitive behavior
Peters-Klimm et al.[73] 2010	197	Case manager-led intervention with 1 individual session followed up by 3 home visits and telephone calls	NYHA-Class, structured assessment of relevant lifestyle, and habits in relation to heart failure	X		X	Chronic care model (framework)
Riegel et al.[71] 2002	358	Nurse-led TC intervention up to 6 months after discharge	Symptoms, knowledge and needs			X	NR
Riegel et al.[65] 2004	88	Peer support intervention with weekly contacts for 3 months	Patient preferences and based on interaction mentor and mentee	X	X	X	Social support model
Riegel et al.[66] 2006	134	Nurse-led TC intervention (same as Riegel 2002 but now targeted to Hispanics)	Symptoms, knowledge, needs, and language/culture.	X	X	X	NR
Shao et al.[114] 2013	108	Research assistant-led intervention with 1 individual visit and 4 TCs within 12 weeks	Self-management behaviors and problems	X			Social cognitive and learning theory
Shearer et al.[115] 2007	87	Nurse-led intervention with 6 telephone calls within 12 weeks after discharge	Needs, goals, and health concerns	X			Theory of Unitary Human Beings
Shively et al. [116] 2005	116	Nurse-led intervention of four group classes and 3 TCs in 4 months	Behavioral goals	X			Information-behavior-motivation model
Shively et al.[77••] 2013	84	Nurse-led intervention of 6 individual face-to-face or TC sessions during 6 months	Activation level	X			Patient activation theory
Sisk et al. [117] 2006	406	Nurse-led intervention with 1 visit and FU TCs for 12 months	Daily sodium intake, medication adherence	X			NR
Wakefield et al.[118] 2008	148	Nurse-led intervention with 14 TCs or videophone contacts within 11 weeks	Symptoms and skills	X			NR
Wongpiriyayothar et al.[119] 2008	93	Researcher-led intervention with 2 home visits and 2 FU TCs within 12 weeks	CHF symptom management, barriers, cooking pattern	X			Conceptual framework of symptom management model developed by Dodd et al. and coaching strategies

^a^Variables used to assess patients and assign them to a tailored intervention component

*NYHA* New York Heart Association, *PCP* primary care physician, *TC* telephone contact/consultation, *FU* follow-up, *NR* not reported

### Tailoring Strategies

The 28 studies included a wide range of patient characteristics which guided tailoring of the content, dose, or mode of the intervention. The most frequently mentioned were HF-related knowledge, self-management behaviors/skills (such as medication adherence, sodium intake, identify symptoms of deterioration, problem solving), barriers for self-management, and patient-reported needs and preferences. Other variables mentioned were environmental factors, health literacy, New York Heart Association (NYHA) class, and activation level.

Tailoring was applied mostly with regard to content, thus addressing different topics based on the assessed patient characteristics. Six interventions customized on the mode of delivery, either changing the language [63••, 64–66] or adapting the literacy level [67], and one study gave a choice in telephone or e-mail contact [68]. Nine interventions varied contact frequency based on needs [66, 69–71], NYHA-class [72, 73], or patients’ preference [63••, 65, 68]. None of the studies reported on tailoring with regard to behavior change techniques.

We found five studies with an intervention designed for and targeted to a specific subgroup. Davis et al. targeted the intervention to HF patients with mild cognitive impairment by focusing on environmental manipulation (e.g., simplifying tasks) and training compensatory strategies (e.g., memory aids) [74]. Barnason et al. targeted their intervention to HF patients at risk for impaired medication adherence (five or more routinely scheduled prescriptions) [67]. Riegel et al. [66] adjusted a previous intervention to Hispanics by integrating cultural values and the possibility of bilingual support. In two subsequent studies, DeWalt et al. targeted their intervention to low literate patients, where the intervention was not only given to the subgroup of low literates but to all HF patients included in the trial [63••, 75].

### Theoretical Models Underlying Tailored Interventions

In 14 of the tailored interventions, theoretical models were used as the underlying mechanism of the intervention. Most often Bandura’s social cognitive theory was used, particularly aiming at increasing patients’ self-efficacy [76]. In one single study, a theoretical model was explicitly used for tailoring. Shively et al. used the activation theory to tailor the intervention based on patients’ initial activation level as measured by the Patient Activation Measure (PAM) [77••]. Patients’ baseline level of activation was assessed as low or medium to high and subsequently individualized goals were set for knowledge (low PAM) or skills and behaviors (medium and high PAM) [77••].

## Patient Characteristics Associated With Self-Management Capacity

A large number of cross-sectional studies have evaluated the association between patient characteristics and varying self-management capacity to identify patients at risk for poor self-management capacity. Although not exhaustive, a summary of the most important identified characteristics is provided below.

In the early 1990s, Connely et al. already developed a conceptual model to increase the understanding of varying self-management capacity, the Model of Self-Care in Chronic Illness (MSSCI) [78]. In chronic disease patients, gender, age, income, education, social support, symptom severity, and comorbidities were initially identified as determinants of self-management capacity [78]. The determinants hypothesized in the MSSCI have been tested in multiple HF populations with relatively inconsistent findings. Rockwell et al. found level of education and symptom severity to explain 10.3 % of the variance in self-management capacity [79]. A subsequent study could not replicate these findings but found that males and patients with less comorbidities were more likely to be good self-managers [30]. Partly similar findings were found by Cameron et al. where older age, male gender, comorbidities, and depression were associated with poor self-management capacity [31]. The association with depression [80] and poor self-management capacity has been replicated in several studies [32, 81]. Besides depression, anxiety and cognitive dysfunction are highly prevalent in patients with HF and seem to complicate engagement in self-management [32, 82, 83]. Performance in self-management also seems to be influenced by experience. Two studies have investigated the relation between the time from diagnosis and showed better scores for experienced patients on self-care maintenance and self-care management scores but surprisingly not on self-care confidence [84, 85]. Other important characteristics that have shown to hamper self-management behaviors in HF are both low literacy and low health literacy. For patients with inadequate literacy, it is more difficult to understand health information and act upon it to perform good self-management [86]. For low health literacy, several studies found it to be independently associated with poor self-management capacity [87–89].

Alongside designating patients with a higher likelihood for poor self-management capacity, decision making on tailored strategies might also be served by getting an in-depth understanding of varying self-management behaviors. Meanwhile, two recent meta-syntheses have been conducted, synthesizing results from a large number of qualitative studies aimed at increasing our understanding on varying self-management capacity [90•, 91•]. The first included 23 studies indicated that disease severity, limited knowledge, comorbidities, cognitive and emotional dysfunction, communication skills, and adverse coping strategies were barriers for adequate self-management capacity. Facilitators were social support, disavowal (healthy denial) coping strategy, trust in health care providers, spiritual beliefs, and optimism [90•]. The most recent meta-synthesis mainly focused on the impact of different contextual factors and included 45 studies. Strachan et al. identified six main types of contextual factors to complicate self-management in HF patients: lack and overload of caregiver support (usually family members), limited social networks, living in rural areas, limited financial capacity, and limited interaction with peers [91•].

Summarizing the great efforts that have been made, we can draw a tentative conclusion that age, gender, (health) literacy, socio-economic class, comorbidities, and emotional and cognitive function are key recurring patient characteristics that are associated with self-management capacity. It is appealing to assume that results from cross-sectional studies might help us to guide targeting or tailoring interventions. Yet, factors associated with self-management capacity are not necessarily those with a higher likelihood for success of self-management interventions. The cross-sectional nature of these studies only provide a snapshot of the association at one time point and give no indication of causal mechanism occurring over time initiated by an intervention.

## Patient Characteristics Associated With Success of a Given Intervention

In contrast to cross-sectional analyses which solely allow for the identification of characteristics associated with self-management capacity, decision making on tailoring can be facilitated by unraveling success of self-management interventions. Quantifying in whom a given intervention is most likely to be successful can be analyzed by identification of effect modification [92, 93]. Effect modification is defined as the difference in the association between the treatment and outcome across different subgroups of patients [94].

Effect modifiers can be identified by stratified analysis where the association between the intervention and outcome is reported for each level of a baseline characteristic. Differences in this association suggest the presence of effect modification [95]. Another method is the measurement of statistical interaction, whereby the statistical significant contribution of the product term between treatment exposure and the baseline characteristic indicates effect modification [94]. To identify patient characteristics modifying success of HF self-management interventions, we could again rely on the selection of randomized trials as reported in the previous section.

Table 2 shows the results of studies evaluating effect modification including the type of analysis and included baseline variables. From the 52 interventions, 12 analyzed the presence of effect modification. The majority focused on demographic variables. None of the studies found differences in success of self-management interventions across different levels of age [40, 42, 50, 63••, 96••] or race [50, 63••, 96••]. This finding is in contrast to well-established associations between these characteristics and self-management capacity. The same accounts for gender differences except for Mårtensson et al. who found subtle gender-specific differences in effectiveness of a nurse-led self-management intervention on SF-36 subscales in the advantage of women [56]. In an additional analysis on self-management behaviors, they showed this could have been mediated by women having a higher tendency to improve adherence to daily weight control [97]. Two separate studies evaluated effect modification in the HART trial and evaluated the impact of low socio-economic class. Although not associated with change in HRQoL [63••], low-income patients (annual family income of <$30,000) receiving a self-management intervention had 44 % longer time to hospitalization or death compared to low-income patients receiving education only [50]. The same direction of association is seen for level of education. Although previous cross-sectional studies (incl. qualitative studies) have shown that low education is associated with poor self-management capacity, additional analysis nested in a large Dutch trial [57] indicates that in fact this group might particularly benefit from the widely validated Chronic Disease Self-Management Program (CDSMP) [98]. The authors found that patients attended <12 years of education gained more improvements in HRQoL compared to their higher educated counterparts. In the same study, cognitive function was identified as an effect modifier of success of CDSMP too, favoring those with a higher baseline cognitive status [98]. The impact of low literacy on success of self-management interventions has been thoroughly investigated by DeWalt and colleagues. Initially, in a relatively small single-center RCT, this research group evaluated the effectiveness of a literacy-sensitive intervention. Although statistically underpowered, they performed a stratified analysis within two levels of baseline literacy and found no indications for effect modification [75]. In a subsequent larger trial (*n* = 605), they examined the difference in effectiveness between a single-session self-management intervention and additional telephonic reinforcement. They reported the impact of age, sex, education, income, ethnicity, NYHA class and literacy, and change in several outcome measures. Only literacy was identified as an effect modifier of the extensive intervention. While emphasized in many studies as a factor hindering successful self-management behavior, having a baseline low/marginal literacy was associated with a higher likelihood for success both in HRQoL [99] and number of hospitalizations [63••].

**Table 2:** Effect modification in longitudinal HF self-management studies

Author, Year	Contrast	Effect modification analyses	Baseline variables analyzed for effect modification	Outcome	Effect modifiers identified
Aldamiz et al.[40] 2007	SM vs. usual care	Stratified analysis	Age, sex, etiology, ejection fraction, original admission due to non-adherence	Combined outcome death/hospitalizations	Admission due to non-adherence = ↓ events
Baker et al. 2011 & DeWalt et al. 2012 [63••, 99]	SM multiple sessions vs. SM single session	Stratified analysis/interaction analysis	Age, sex, education, income, race, NYHA class, literacy	HRQoL	low/marginal literacy = ↑ gain in HRQoL
				Self-efficacy	None
				Knowledge	None
				Self-care behaviors	None
				Hospital admissions	Low/marginal literacy = ↓ events
Bocchi et al.[42] 2008	SM vs. usual care	Stratified analysis	Age, sex, NYHA class, ethnicity, DM, etiology	Combined outcome; death/hospitalizations	NYHA I/II = ↓ events
DeWalt et al.[75] 2006	SM vs. usual care	Stratified analysis	Literacy	HRQoL	None
				Combined outcome; death/hospitalizations	None
Heisler et al.[46••] 2013	SM vs. usual care	Stratified analysis	Sex	TTE combined outcome; death/hospitalizations	None
Jaarsma et al.[100] 2010	SM vs. usual care	Interaction analysis	Depression	Combined outcome; death/hospitalizations	Depression = ↑ events
				Combined outcome; TTE death/hospitalizations	Depression = ↓ time to event
				Death	Depression = ↑ events
				TTE death	Depression = ↓ time to event
				Hospitalizations	Depression = ↑ events
				TTE hospitalizations	None
Laramee et al.[48] 2003	SM vs. usual care	Stratified analysis	Distance from hospital, care from local specialist	Hospital admissions	Shorter distance from hospital = ↓ events; care from local specialist = ↓ events
Martensson et al.[56] 2005	SM vs. usual care	Stratified analysis	Sex	Generic QoL	Female = ↑ generic QoL
Powell et al. 2010 & Grady et al. 2014[50, 96••]	SM vs. enhanced education	Interaction analysis	Age, sex, education, income, race, 6 MWT, comorbidities, systolic function, drug adherence	Combined outcome; death/hospitalizations; combined outcome; TTE death/hospitalization	None
					Low income (< $30,000/year) = ↓ time to event
			Age, sex, race, education, income, depression, NYHA class	HRQoL	NONE
Shively et al.[77••] 2013	SM vs. usual care	Interaction analysis	PAM	Hospital admissions,	Low or high PAM scores = ↓ events
				ED visits	None
				PAM	Medium PAM scores = ↑ gain in PAM
Smeulders et al.[98] 2010		Interaction analysis	Age, sex, time from diagnosis, NYHA class, comorbidities, education, living status, cognition, employment	HRQoL	Higher cognitive function = ↑ gain in HRQoL lower education = ↑ gain in HRQoL

*HRQoL* Health-related quality of life, *PAM* patient activation Measure, *ED* emergency department, *6MWT* 6-min walking test, *NYHA* New York Heart Association, *TTE* time to event, *DM* diabetes mellitus

Five studies [40, 50, 57, 63••, 96••] evaluated the effectiveness of self-management intervention within strata of HF-specific characteristics such as severity (NYHA class, ejection fraction, time from diagnosis) and ischemic etiology. Only Bocchi et al. found that in patients with baseline NYHA I–II, a program with repetitive education and telephone monitoring resulted in a larger risk reduction on the composite outcome hospitalization or death [42].

As a reflection of disease complexity, two studies have evaluated the impact of comorbidities. Although operationalization of comorbidities varied, no indications for effect modification were observed [50, 57]. Yet, specific comorbidities do seem to hinder the success of self-management. In addition to the negative influence of low cognitive function, as indicated earlier, baseline depression might also lead to less successful outcomes of self-management interventions. While no effect modification was identified for HRQoL in the HART trial [96••], interaction analysis in the COACH trial showed that the intervention was only effective on time to death/readmission and reduced mortality rates in patients without depression [100].

Few studies have analyzed whether baseline self-management competences can influence success of interventions. One might assume that the initial level of HF-specific self-management behaviors and known mediators of change in self-management capacity such as self-efficacy and activation determine the didactic room for improvement. This is supported by the interaction analysis nested in a Spanish RCT where they analyzed a broad range of potential effect modifiers. In a subgroup of patients whose original admission was attributed to non-adherence, the risk reduction for combined readmission or death rate was significantly greater [40]. Shively et al. studied the impact of baseline levels of activation, as measured by the PAM on success of a self-management intervention [77••]. Their results indicated that moderately activated patients were the ones with the highest likelihood for a positive change in the PAM, while in low and highly activated patients, the intervention resulted in a higher reduction in hospitalizations. Interpretation of these results is complicated by the fact that the intervention was tailored based on patients’ initial level of activation [77••].

In summary, age, ethnicity, gender, number of comorbidities, and disease severity do not seem to influence the success of self-management interventions. Low education, low income, low literacy, and low baseline self-management capacity seem to facilitate success, while depression and cognitive dysfunction seem to hinder success of self-management interventions.

## Clinical Implications

To curb the complexity of HF and to reduce its impact on patients and society, a multi-faceted approach is needed, in which patients have an important responsibility in determining the course of their disease. Improved self-management capacity contributes to better HF-related outcomes and reduction in hospitalizations and mortality. Interventions aimed at supporting patients in increasing these competences have shown to be successful, however, not in all patients. To optimize effectiveness of those interventions prompts the use of better targeted or tailored interventions. It is likely that adaptive interventions, combining the identification of certain subgroups and subsequent tailored solutions will result in higher effect sizes. However, within the large number of available trials, the degree to which interventions are explicitly tailored is marginal.

For future interventions and to support clinical practice, we have summarized the results of many scientific efforts to identify patient characteristics that are clinically relevant to guide targeting and tailoring. For age, gender, ethnicity, disease severity, and number of comorbidities, substantial evidence is available that these factors do not seem to influence the degree of success of self-management interventions. Other characteristics initially identified as factors potentially hindering effective self-management in longitudinal studies are in fact indicators of those patients with the highest likelihood for improvement. Knowledge of these effect modifiers gives rise to formally include these factors in the screening process, especially when more efficient allocation of resources is indicated. Patients with low literacy, low education, and low income at baseline have shown to be suitable candidates for self-management support. It can be expected that more tailored approaches can even further boost effect sizes (e.g., (health) literacy sensitive interventions). Effective guidelines are available to provide patients with additional support, adapt communication strategies, and educational content and materials [86, 101]. Low-income patients already seem to benefit from current self-management interventions, yet policy-makers should stay vigilant to prevent financial factors from hindering the effects of self-management.

Another important aspect is the baseline assessment of self-management capacity including important mediators of behavior change such as self-efficacy, knowledge, adherence, and activation. A limited number of studies indicate that initial poor self-management behaviors do not hinder but instead represent those with a high didactic potential for improvement.

Current evidence of effect modification in clinical trials indicates that certain characteristics do hamper the likelihood for success of self-management interventions. Cognitive dysfunction and depression are highly prevalent comorbidities in HF, and both seem to have negative impact on the effectiveness of self-management interventions. Therefore, formal screening at baseline for these factors is imperative. For cognitive dysfunction, both learning and subsequently performing self-management are highly challenging. In current available trials, interventions seldom formally screen for cognition and do not seem to be sensitive in sufficiently tackling the influence of cognition on increasing self-management competences. It seems sensible that interventions in this subgroup of patients need to be extended with increased assistance, allocation of caregivers, cognitive training, and environmental manipulations, i.e., by simplifying tasks, providing external cues, or prompts to initiate action [74]. Given the high prevalence in the HF population, the impact of depression on success of self-management is disturbing. Although little evidence is available on tailored strategies in this subgroup, it seems useful to incorporate screening of depressive symptoms at enrollment and to consider cognitive behavioral therapy or to add antidepressants [102].

## Research Implications

Extensive scientific work has been done to increase our understanding in whom self-management is problematic and in whom self-management interventions are most effective. Yet, the interplay between patient characteristics, self-management capacity, and the likelihood for success of interventions is still unclear. In our quest to establish tailored HF self-management, we should slowly abandon from cross-sectional analyses, since many studies already have been performed, and they have provided limited information on the causal mechanisms towards success. Instead, decision making on tailored strategies is served by comprehensive identification of effect modifiers from longitudinal studies.

Understanding the variance in effectiveness among subgroups is complicated by the heterogeneity in available intervention strategies. Substantial variations in intervention components, mode of delivery, and dose hamper answering “what works in whom,” the key towards effective tailoring of future interventions. Increased scientific efforts are needed to validate the modifying impact of currently known characteristics such as (health) literacy, education, income, cognitive, and emotional status across different treatment strategies. In addition, more knowledge is needed on the extent to which initial self-management capacity and levels of key baseline behavioral mediators reflect the potential for improvement. Stratified analysis in well-powered randomized trials, preferably with multiple treatment arms, can help us to further unravel the causal mechanism between patient and intervention characteristics. Additionally, data from longitudinal routine-care cohorts and an ongoing Individual Patient Data meta-analysis [35•] can help us to untangle success of self-management as a bridge towards more individually tailored strategies.

Available trials on HF self-management have not always clearly described how tailoring was applied. The minority of studies which explicitly described the tailoring strategy generally limited tailoring to individualizing content. Guided by the accumulating knowledge in this field, future studies are needed to study the added value of tailoring interventions in terms of behavioral change techniques [103], mode of delivery (i.e., regular consultations vs. e-Health solutions), and intensity. A promising design to this purpose is the use of adaptive interventions designs, whereby the type or dosage of the intervention is adapted based on patient characteristics and the treatment is adjusted repeatedly over time [104]. These interventions use individual differences between participants to achieve the best possible outcome, both by augmenting an intervention for a non-responsive participant or diminishing treatment for a responsive participant in order to reduce cost or participant burden. Sequential, multiple assignments, randomized trials (SMART) can be used to test the decision rules for an adaptive intervention [105].

## Conclusion

Although embedded in HF guidelines, effectiveness of self-management interventions has not yet reached its full potential. Effectiveness can be substantially optimized when interventions are better tailored to individual patients. Currently, available trials did individualize the intervention to a certain extent; however, this was rarely applied through explicit identification of certain subgroups and subsequent provision of tailored solutions. In this review, we synthesized essential literature which can serve as building blocks for future development of tailored self-management approaches. The results show that we have to redirect our efforts: several characteristics previously considered to be associated with poor self-management capacity (age, gender, ethnicity, disease severity, number of comorbidities) actually do not affect effectiveness of a given intervention. Other factors such as low income, low literacy, low educational level, and low baseline self-management capacity in fact point at those patients with the largest potential for improvement. Identifying those patients with a high likelihood for success can increase effectiveness of interventions. Particular emphasis should be placed on pro-active screening and attempts to develop and validate tailored strategies for patients with cognitive or emotional dysfunction. Meanwhile, scientific efforts are needed to unravel success of self-management interventions and the added value of tailoring.
